# Supplementary material for: Molecular ferroelectric with low-magnetic-field magnetoelectricity at room temperature
Source: Nat Commun. 2024 Jun 3;15:4702. doi: 10.1038/s41467-024-49053-y (PMC11148071; doi:10.1038/s41467-024-49053-y)
Supplement: Supplementary file 1 — Supplementary Information [file 41467_2024_49053_MOESM1_ESM.pdf]

Supporting Information

Molecular ferroelectric with low-magnetic-field magnetoelectricity at room temperature

Zhao-Bo Hu[1,2], Xinyu Yang[3], Jinlei Zhang[4], Ling-Ao Gui[2], Yi-Fan Zhang[2], Xiao-Dong Liu[1], Zi-Han Zhou[1], Yucheng Jiang[4], Yi Zhang[5], Shuai Dong[3] & You Song[1]

[1] State Key Laboratory of Coordination Chemistry, School of Chemistry and Chemical Engineering, Nanjing University, Nanjing 210023, China

[2] Chaotic Matter Science Research Center, Department of Materials, Metallurgy and Chemistry & Jiangxi Provincial Key Laboratory of Functional Molecular Materials Chemistry, Jiangxi University of Science and Technology, Ganzhou 341000, China

[3] Key Laboratory of Quantum Materials and Devices of Ministry of Education, School of Physics, Southeast University, Nanjing 211189, China

[4] Jiangsu Key Laboratory of Micro and Nano Heat Fluid Flow Technology and Energy Application, School of Physical Science and Technology, Suzhou University of Science and Technology, Suzhou, 215009, China

[5] Institute for Science and Applications of Molecular Ferroelectrics, Key Laboratory of the Ministry of Education for Advanced Catalysis Materials, Zhejiang Normal University, Jinhua, 321004, China

Correspondence and requests for materials should be addressed to J.L.Z. (email: zhangjinlei@usts.edu.cn) Y.Z. (email: yizhang1980@seu.edu.cn), S.D. (email: sdong@seu.edu.cn) or to Y.S. (email: yousong@nju.edu.cn).

Supplementary Table 1 | Crystallographic data. Structural data for (TMCM)[$FeCl_4$] at 298 K obtained at zero field and 10 kOe magnetic field. The magnetic field is applied along the *c*-axis. The lattice constants of (TMCM)[$FeCl_4$] become lengthening along the *c*-axis while become shorter in the *ab* plane.

	H = 0 kOe	H = 10 kOe	$\Delta x/x$
T (K)	298	298	
Crystal System	Monoclinic	Monoclinic	
Space Group	*C*m	*C*m	
a (Å)	13.0043(7)	13.0808(5)	5.9×10^{-3}
b (Å)	14.8164(7)	14.7898(5)	-1.8×10^{-3}
c (Å)	6.4817(3)	6.5327(2)	7.9×10^{-3}
β (°)	98.881(4)°	98.706(4)°	-1.8×10^{-3}
V (Å^3)	1233.90(11)	1249.27(8)	12×10^{-3}

Supplementary Table 2 | Crystal structure data. Bond distances (at 298 K) involving the Fe^{3+} at 298 K obtained at zero field and 10 kOe magnetic field along the *c*-axis. The volume of [$FeCl_4$]$^-$ anion becomes smaller under field.

Bond length	H = 0 kOe	H = 10 kOe	$\Delta x/x$
Fe(2)-Cl(5)	2.182(3)	2.178(3)	-1.8×10^{-3}
Fe(2)-Cl(6)	2.185(2)	2.187(2)	0
Fe(2)-Cl(4)#1	2.1958(16)	2.1885(14)	-3.3×10^{-3}
Fe(2)-Cl(4)	2.1958(16)	2.1885(14)	-3.3×10^{-3}
Fe(1)-Cl(3)	2.191(3)	2.190(2)	0
Fe(1)-Cl(2)	2.191(3)	2.191(2)	0
Fe(1)-Cl(1)#2	2.2017(16)	2.1936(14)	-3.7×10^{-3}
Fe(1)-Cl(1)	2.2017(16)	2.1936(14)	-3.7×10^{-3}

Supplementary Table 3 | Crystal structure data. Bond angles involving the Fe^{3+} at 298 K obtained at zero and 10 kOe magnetic field along the *c*-axis.

Bond angle	$H = 0$ kOe	$H = 10$ kOe	$\Delta\theta/\theta$
Cl(5)-Fe(2)-Cl(6)	108.24(16)	108.94(17)	6.5×10^{-3}
Cl(5)-Fe(2)-Cl(4)#1	108.35(9)	108.30(9)	0
Cl(6)-Fe(2)-Cl(4)#1	110.59(7)	110.14(6)	-4.1×10^{-3}
Cl(5)-Fe(2)-Cl(4)	108.35(9)	108.30(9)	0
Cl(6)-Fe(2)-Cl(4)	110.59(7)	110.14(6)	-4.1×10^{-3}
Cl(4)#1-Fe(2)-Cl(4)	110.64(10)	110.96(9)	3×10^{-3}
Cl(3)-Fe(1)-Cl(2)	107.91(13)	108.11(13)	0
Cl(3)-Fe(1)-Cl(1)	109.31(7)	109.27(7)	0
Cl(2)-Fe(1)-Cl(1)	110.14(7)	109.76(6)	-3.5×10^{-3}
Cl(3)-Fe(1)-Cl(1)#2	109.31(7)	109.27(7)	0
Cl(2)-Fe(1)-Cl(1)#2	110.15(7)	109.76(6)	-3.5×10^{-3}
Cl(1)-Fe(1)-Cl(1)#2	109.98(10)	110.63(9)	5.9×10^{-3}

Supplementary Table 4 | DFT calculated basic physical properties. Energies of four magnetic orders (in units of meV/f.u.) and optimized lattice constants (in units of Å) of (TMCM)[$FeCl_4$]. The energy of G-AFM state is taken as the reference. The experimental (Exp) one is shown for comparison. *M* is the magnetic moment (in units of μ_B).

Order	Energy	*a*	*b*	*c*	*M* (Fe)
bulk (Exp)	—	13.004	14.816	6.482	—
G-AFM	0	12.769	14.758	6.420	±4.06
C-AFM	0.69	12.768	14.761	6.419	±4.06
A-AFM	0.67	12.775	14.757	6.419	±4.06
FM	1.61	12.789	14.752	6.415	4.06

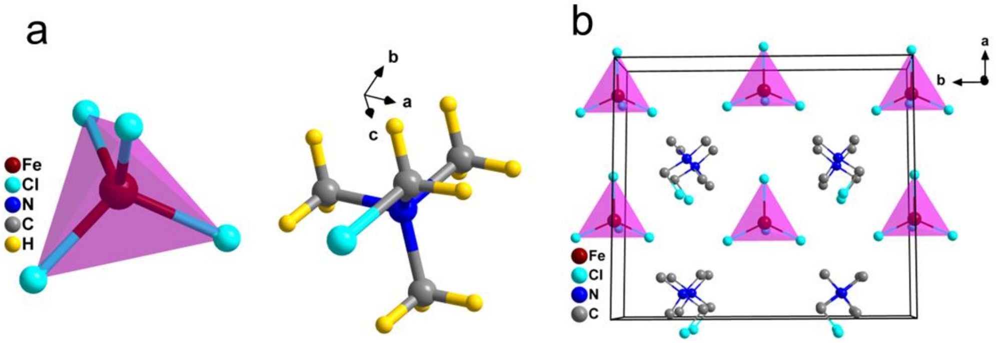

Supplementary Fig. 1 | The crystal structure. a The molecular structure of (TMCM)$[FeCl_4]^-$, consisted by the inorganic tetrahedron and TMCM molecule. The C–Cl bond is much longer than the C–H bond, and the C–Cl bond length is 1.737 Å. This should lead to a significant decrease of the sphericity of the halogenated cations. **b** The packing of (TMCM)$[FeCl_4]$.

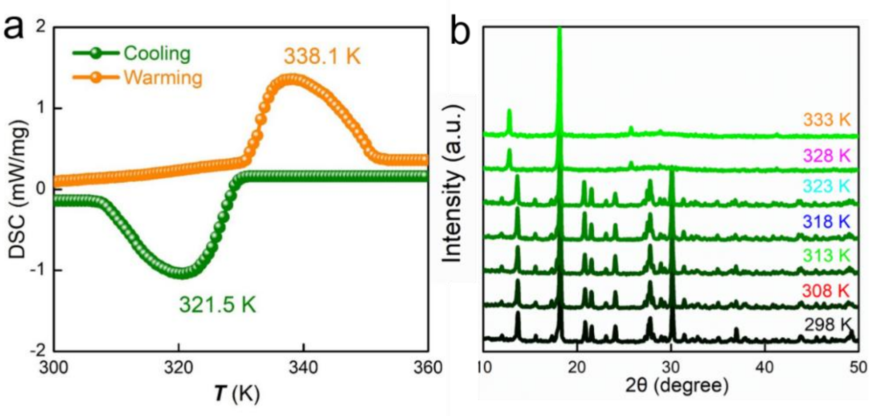

Supplementary Fig. 2 | DSC and Rietveld refinement of powder X-ray diffraction (PXRD). **a** DSC curves in a cooling-heating runs indicating phase transformation properties. **b** The XRD spectrum of molecular material (TMCM)[$FeCl_4$] under different temperatures range from 298 to 333 K. The XRD spectra shows obvious changes when the temperature rises to 328 K, confirming the phase transition observed in DSC measurement.

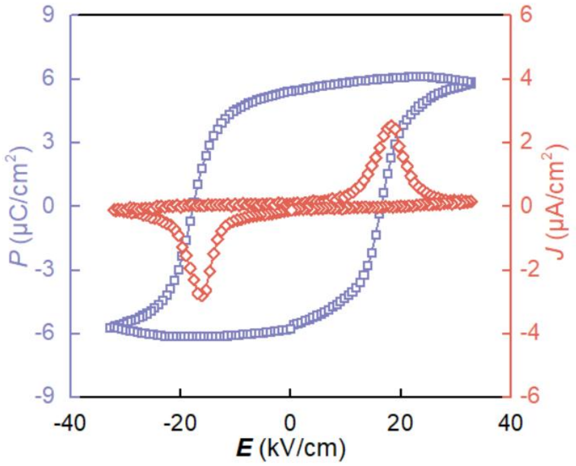

Supplementary Fig. 3 | *P-E* curves. The *P*-*E* (blue) and *J*-*E* (red) hysteresis loops of (TMCM)[$FeCl_4$], measured at 310 K along the *c*-axis.

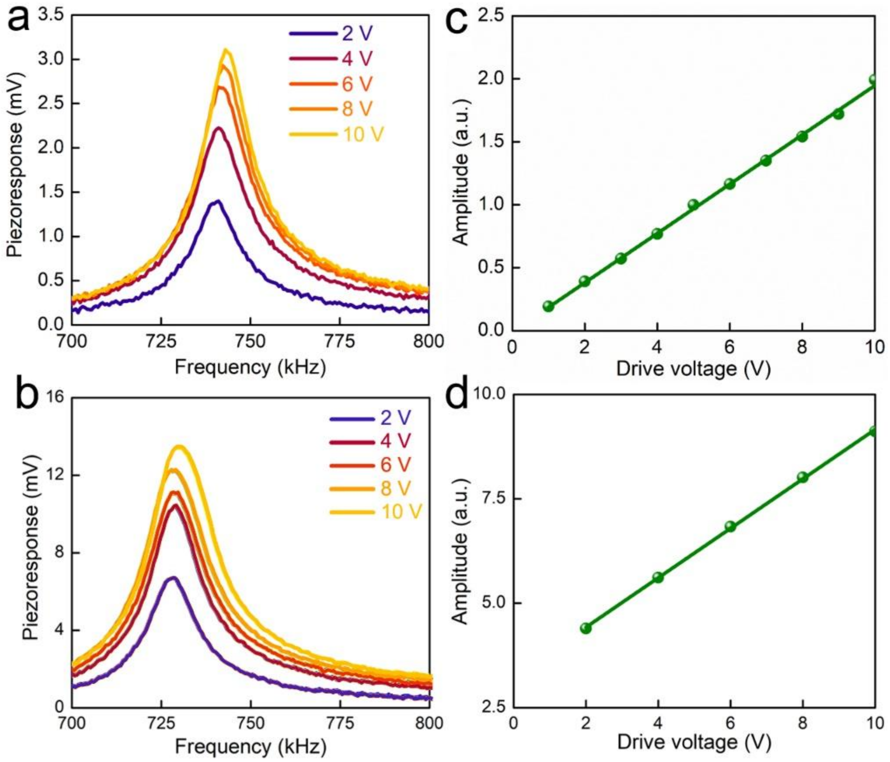

Supplementary Fig. 4 | Piezoelectric properties. a,**b** Piezoelectric properties of PFM resonance peaks in (TMCM)[$FeCl_4$] along the *a*-axis (**a**) and the *c*-axis (**b**), respectively. **c**,**d** The effective piezoelectric coefficient along the *a*-axis (**c**) and the *c*-axis (**d**), respectively. Typical resonance curves were excited in (TMCM)[$FeCl_4$] using a PFM probe with voltage from 2 to 10 V at room temperature. Furthermore, to confirm that the response comes from piezoelectricity, we performed amplitude-drive voltage measurements. Such measurements have been performed over a series of drive voltages up to 10 V, showing good linearity. Such good linearity indicates that the response is induced by intrinsic piezoelectricity.

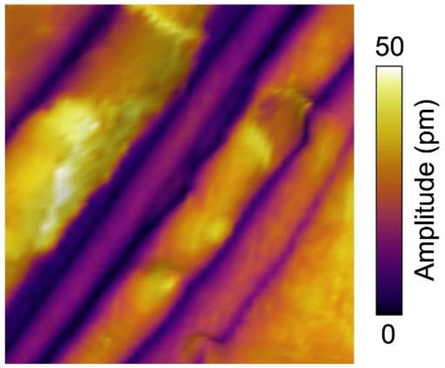

Supplementary Fig. 5 | PFM properties. **a** Vertical PFM amplitude image (1.5×1.5 μm^2) overall morphology showing spike-like ferroelectric domain.

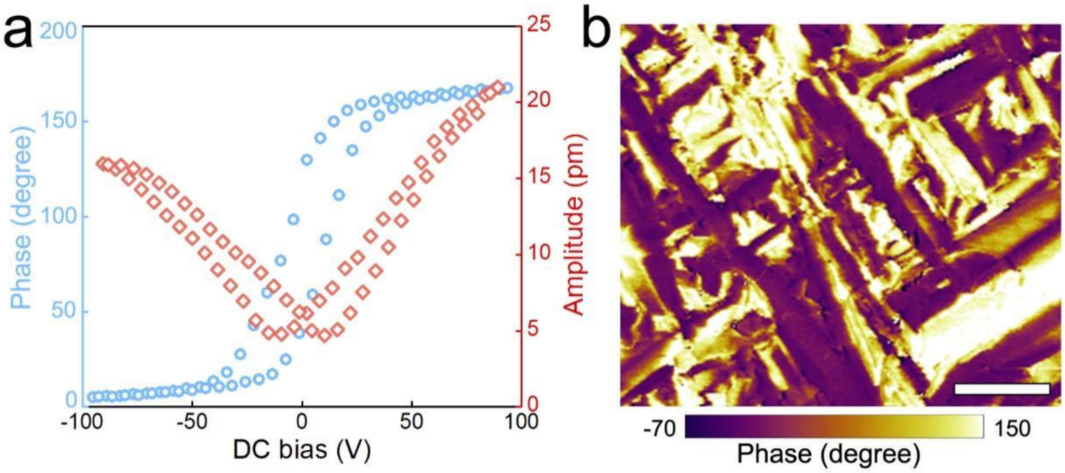

Supplementary Fig. 6 | PFM properties. **a** Phase-voltage hysteresis (blue) and amplitude-voltage butterfly loop (red) of the TMCM[$FeCl_4$] acquired by PFM along the *c*-axis. **b** Phase images showing spike-like ferroelectric domain along the *c*-axis (scale bar 10 μm).

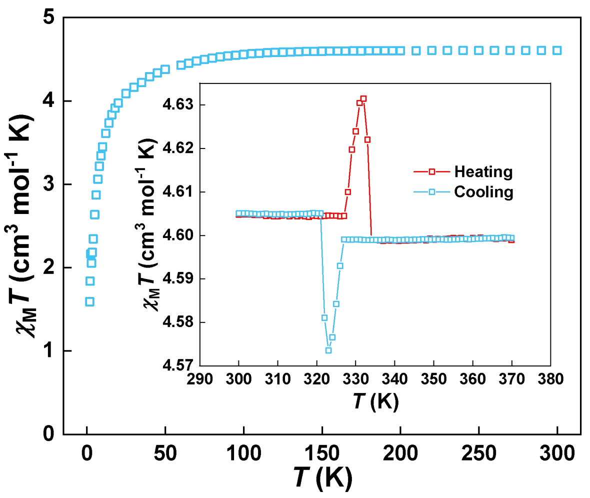

Supplementary Fig. 7 | Magnetic properties. Temperature derivative of the magnetic susceptibility of (TMCM)[$FeCl_4$] confirming the phase transition at 323 K. Inside: heating represent for heating measurement and cooling represent for cooling measurement.

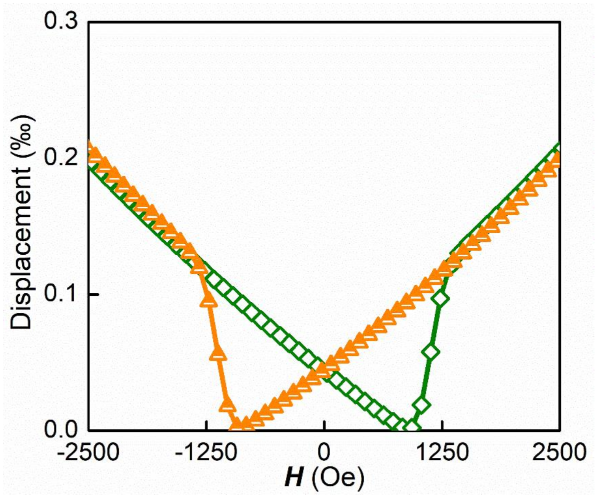

Supplementary Fig. 8 | Magnetostriction properties. Magnetostriction as a function of magnetic field at 300 K measured on a single crystal (TMCM)[$FeCl_4$] along the *a*-axis. We can observe the magnetostriction loop vs magnetic field. The olive curve represents the results of the field up measuring, while the orange one represents the results of the field down measuring.

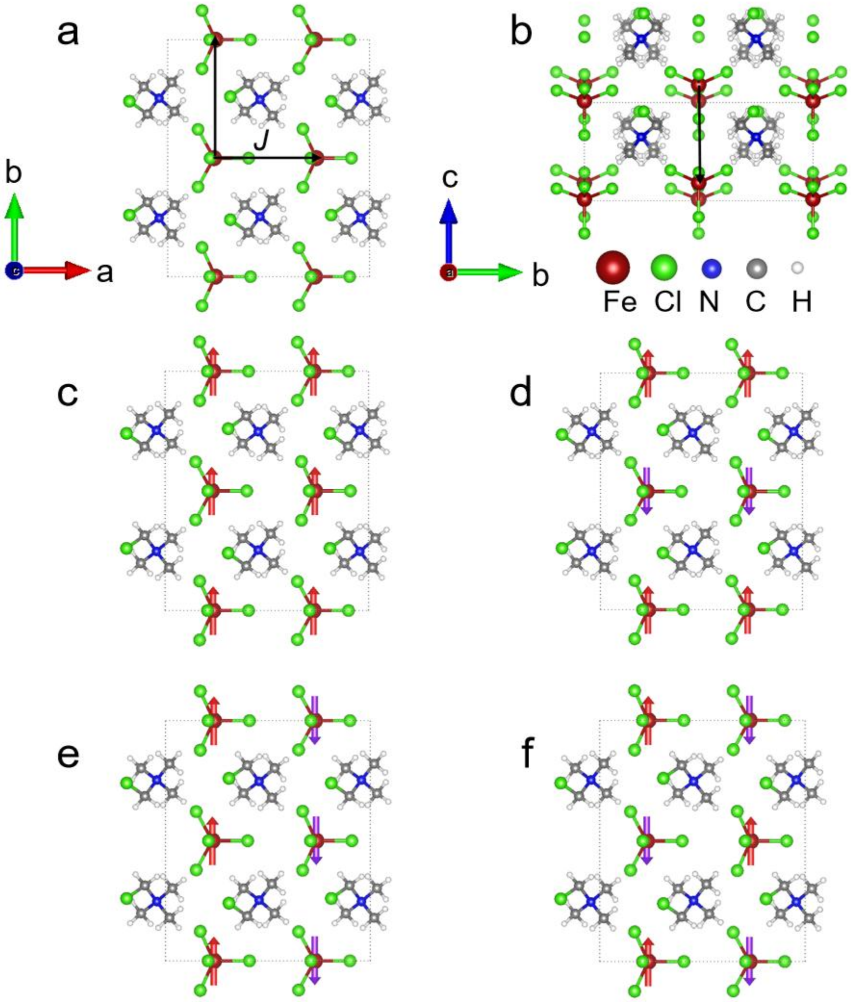

Supplementary Fig. 9 | Possible magnetic orders used in DFT calculation. **a,b** The top (**a**) and side (**b**) views of (TMCM)[$FeCl_4$]. Exchange J is also indicated. Schematic of four most possible magnetic orders: **c** Ferromagnetism (FM). **d** A-type antiferromagnetism (A-AFM). **e** C-type antiferromagnetism (C-AFM). **f** G-type antiferromagnetism (G-AFM). Red arrows: spin up; Purple arrows: spin down.

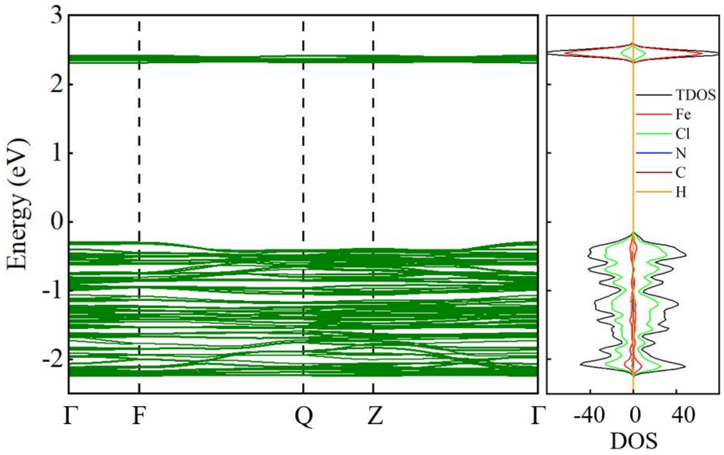

Supplementary Fig. 10 | DFT electronic structures. Band structure and density of states (DOS) of the (TMCM)[$FeCl_4$].

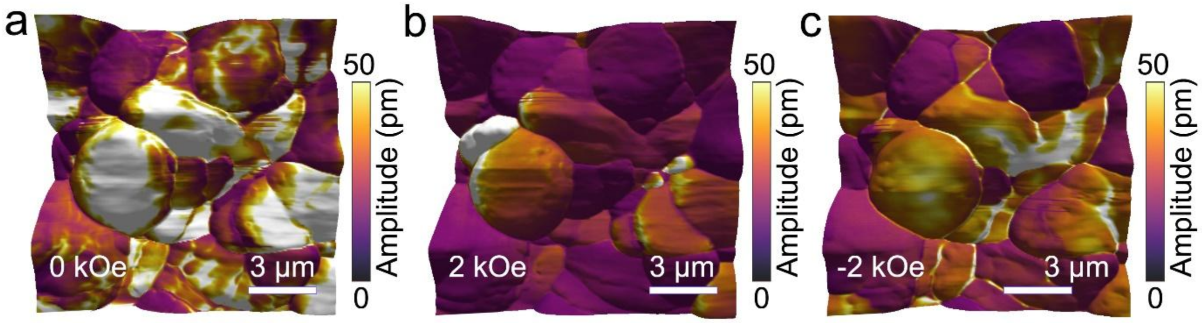

Supplementary Fig. 11 | Ferroelectric domain under different magnetic field. **a** Vertical PFM responses at in-plane $H = 0$ Oe showing local ferroelectric domain. **b**,**c** Vertical ferroelectric domains redistributed at in-plane $H = 2$ kOe (**b**) and $H = -2$ kOe (**c**), respectively.

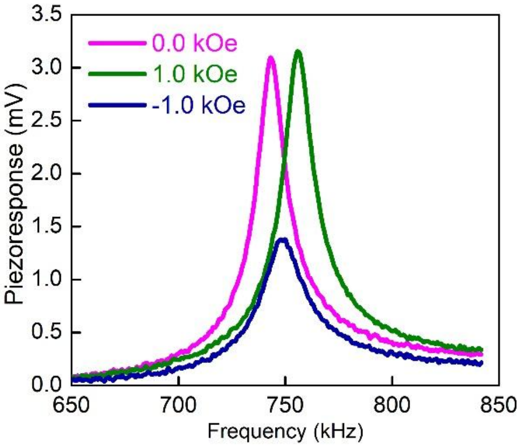

Supplementary Fig. 12 | Amplitude-drive voltage under different magnetic field. Magnetoelectric coupling properties of PFM resonance peaks at drive voltage of 2 V under different external magnetic field. The peak value shows obviously changes under different magnetic field. Furthermore, to confirm that the response comes from magnetoelectric coupling, we performed amplitude-drive voltage measurements in Fig. 3d. Such measurements have been performed over a series of drive voltages up to 10 V under different magnetic field, all showing good linearity. The slope decreases under the external magnetic field of both ±2 kOe. As the relative magnitude of the piezoelectric coefficient (d_{oop}) can be estimated from the slope of the curves. Therefore, the piezoelectric coefficient of (TMCM)[$FeCl_4$] is suppressed obviously under ±2 kOe. The piezoelectric coefficient under -2 kOe shows 50% smaller than the one without magnetic field.

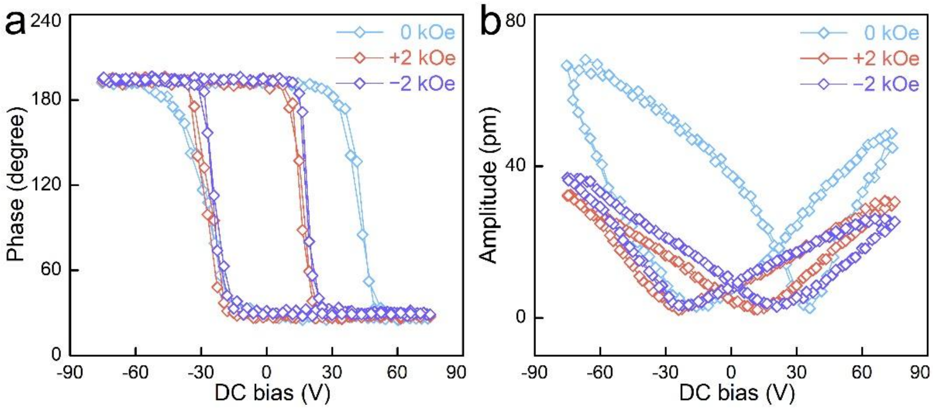

Supplementary Fig. 13 | Piezoresponse hysteresis loops under different magnetic field. **a,b** SS-PFM OFF-filed phase (**a**) and amplitude (**b**) loops under different magnetic field, leading to changes in their asymmetry, amplitude, and coercive fields.

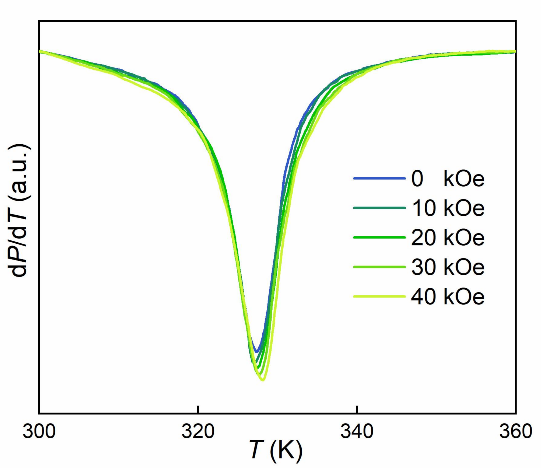

Supplementary Fig. 14 | Temperature dependence of dP/dT under different magnetic field. Differential of polarization (d*P*/d*T*) along the *a*-axis under various external magnetic fields from 0 kOe to 4 kOe.
